# Fatigue Performance of Medical Ti6Al4V Alloy after Mechanical Surface Treatments

**DOI:** 10.1371/journal.pone.0121963

**Published:** 2015-03-30

**Authors:** Robert Sonntag, Jörn Reinders, Jens Gibmeier, J. Philippe Kretzer

**Affiliations:** 1 Laboratory of Biomechanics and Implant Research, Clinic for Orthopedics and Trauma Surgery, Center for Orthopedics, Trauma Surgery and Spinal Cord Injury, Heidelberg University Hospital, Heidelberg, Germany; 2 Institute of Applied Materials, Karlsruhe Institute of Technology (KIT), Karlsruhe, Germany; Université de Technologie de Compiègne, FRANCE

## Abstract

Mechanical surface treatments have a long history in traditional engineering disciplines, such as the automotive or aerospace industries. Today, they are widely applied to metal components to increase the mechanical performance of these. However, their application in the medical field is rather rare. The present study aims to compare the potential of relevant mechanical surface treatments on the high cycle fatigue (R = 0.1 for a maximum of 10 million cycles) performance of a Ti6Al4V standard alloy for orthopedic, spinal, dental and trauma surgical implants: shot peening, deep rolling, ultrasonic shot peening and laser shock peening. Hour-glass shaped Ti6Al4V specimens were treated and analyzed with regard to the material’s microstructure, microhardness, residual stress depth profiles and the mechanical behavior during fatigue testing. All treatments introduced substantial compressive residual stresses and exhibited considerable potential for increasing fatigue performance from 10% to 17.2% after laser shock peening compared to non-treated samples. It is assumed that final mechanical surface treatments may also increase fretting wear resistance in the modular connection of total hip and knee replacements.

## Introduction

Titanium as an engineering material was first extracted to a relevant extent in the early decades of the 20^th^ century from mineral sources by new metallurgy and has, since then, been used for military applications in particular. Some years later between the First and Second World Wars, orthopedic surgeons reported early satisfactory results regarding the material’s strength and corrosion resistance [1]. During the 1950s and 1960s, Leventhal and Brånemark called for its use in medical implants, as they described titanium’s superior ability to avoid corrosion within the human body and the phenomena of osseointegration [2, 3]. Today, titanium and its alloys are the standard biomaterials for medical long-term applications, especially those that require bony ingrowth for fixation of the implant material. This is not only due to its superior biocompatibility compared to other metals, but also to the relatively high mechanical strength that makes it suitable for load-bearing applications, e.g. cementless orthopedic prostheses, dental and trauma surgery implants, such as plates, screws or intramedullary nails.

**Fig 1:**
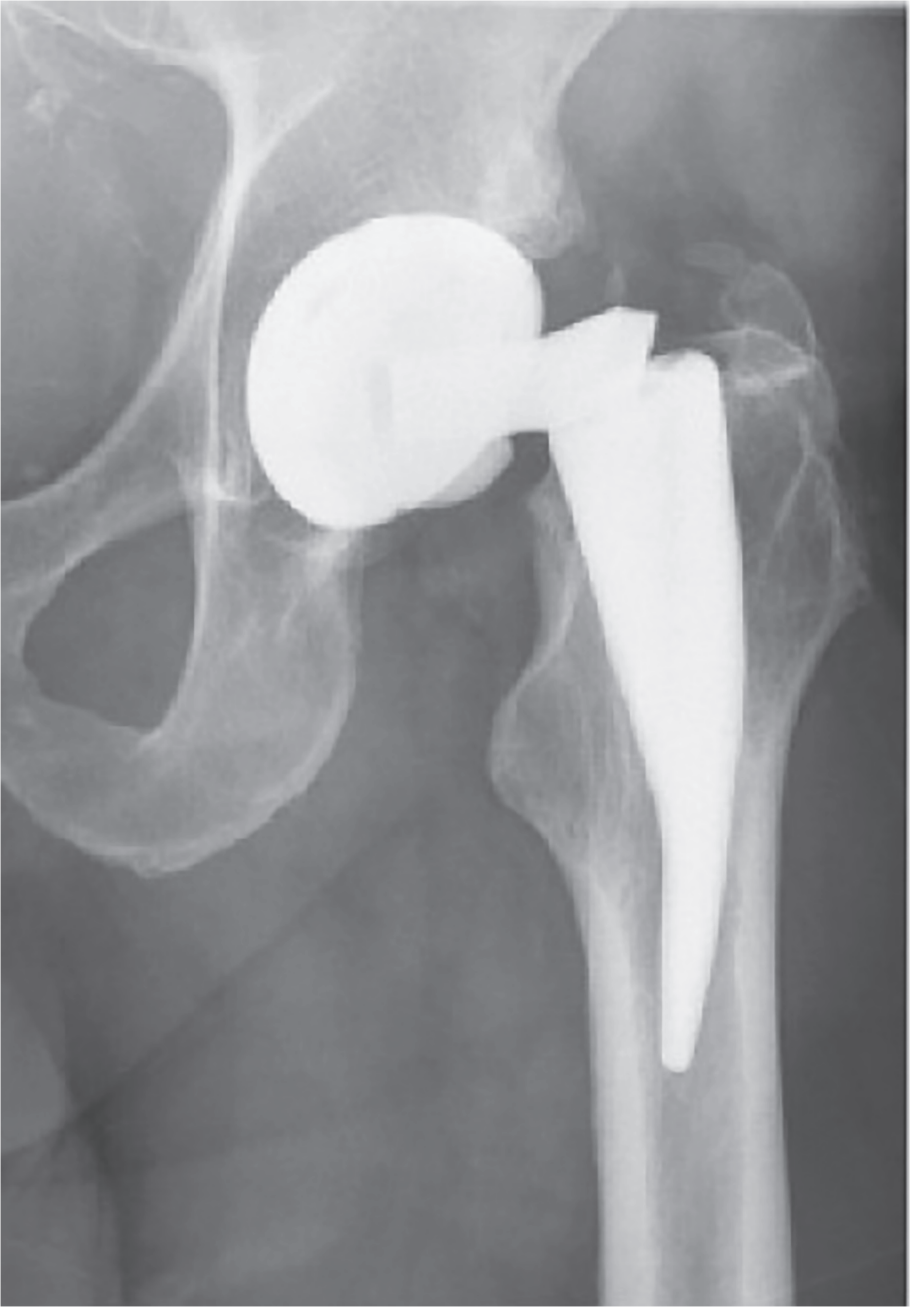
Example of Ti6Al4V implant failure in cementless total hip prosthesis [4].

Besides the medical device alerts and recalls issued by the U.S. Food and Drug Administration (FDA) following an unexpected high failure rate of specific implant systems, there are several cases in literature that have reported fractured titanium or titanium alloy components (Fig 1), even though overall long-term results of these implants are still very good [4–15]. These failures are mainly due to the highly challenging boundary conditions for implant designers. Realization of the implant’s function, within the naturally limited space that is available for implantation, is often linked to a sophisticated design and modular set-up, which further affects overall implant strength. Besides the need for additional surgical intervention, implant failure remains a very painful and traumatic experience for the patient. In most cases, a metal fracture is accompanied by a certain bony defect followed by a complex surgical reconstruction and rehabilitation procedure.

Statistical evaluations on the concrete number of implant fractures have not been reported in the literature so far. However, national registry data for hip and knee arthroplasty are available that differentiate between fractures occurring in the periprosthetic bone and those of the implant material, e.g. the stem of a cementless endoprostheses [16, 17]. Even though the data does not always explicitly indicate which component is affected, the total number of revisions due to metallic failure is less than 1%. The overall survival rates of implant systems for trauma, dental or spine surgery are not available. Although the total number of failed implants of one system may be small, implant fracture always has an economic effect: restricted use of such a system up to the system’s recall from the market followed by legal consequences and an adverse impact in terms of image for the manufacturer.

Most frequently, failure takes place as a result of high tensile stresses at the surface or around notches, e.g. drill holes in intramedullary nails or plates [18]. Exceeding the critical stress within the thin (1.8–17 nm) titanium dioxide (TiO_2_) surface layer may lead to small, superficial micro cracks followed by a fast repassivation [19]. This effect takes place continuously and may even be promoted by the corrosive environment (oxidative wear). In that context, it is known that physiological loading induces unexpected, high cyclic maximum stresses during daily activities [20]. This should be far below the material’s critical strength, but may become relevant after a high total number of cycles over its lifetime (high cycle fatigue, HCF). Even the microcracks within the material, which grow at slow per-cycle velocities (about 10^–10^ to 10^–9^ m/cycle), can propagate to failure throughout course of the implant’s lifetime [21].

Implant modularity was first introduced for total hip replacements by the Russian orthopedic surgeon Konstantin Sivash in the 1960s and it became more popular towards the end of the century (S-ROM, DePuy Orthopaedics Inc., Warsaw, Indiana, USA) [22, 23]. Today, there are a large number of primary and revision implant systems which follow the concept of modularity, reducing stock holding and increasing surgeons’ options during implantation. In that context, unexpected high fracture rates were reported for some implant systems which failed due to increased fretting corrosion at the modular interface [4, 24, 25]. Due to micromotions within the crevice of a taper junction, the thin TiO_2_ layer repeatedly fractures and repassivates. The TiO_2_ layer consequently consumes oxygen resulting in a local decrease of the pH value and an increase in chloride content [26]. These conditions further promote crevice corrosion as repassivation is locally inhibited.

From a practical point of view and so that the increased demands of patients can be addressed, implant manufacturers must ask themselves what they can do to increase the fatigue strength of their components. This applies, in particular, to those that have a sophisticated design based on their function: (a) *Patient*: the external load on the implant may be decreased, e.g. by using crutches, which has a direct impact on the patient’s quality of life and is clearly not a permanent solution. (b) *Design*: the implant’s design may be altered to avoid high stress peaks within the material, e.g. by reducing sharp edges. This approach is particularly important when using titanium or titanium alloys as a notch sensitive material, but it is also limited by the available space and the implant’s function. (c) *Material*: according to the field of application, new high-strength materials may be applied, e.g. high-alloyed steels such as CoCrMo. However, they must not only withstand high stresses but also must be appropriate in terms of biocompatibility. In many cases, such as cementless arthroplasty, titanium and its alloys are the only standard material with suitable clinical long-term results. This is certainly the reference material for further developments, such as new titanium based alloys or carbon fiber reinforced PEEK [27]. (d) *Final surface modifications*: beside thermal treatments, mechanical surface treatments enhance the fatigue and cyclic deformation behavior of titanium alloys and have already been applied in parts within the scope of biomedical applications [28, 29]. From other engineering disciplines, they are known to strengthen the substrate without changing the material’s composition [30]. The overall positive effect of the mechanical surface treatments on the fatigue endurance can be associated with the work hardening, i.e. the high dislocation densities and the compressive residual stresses induced in the near surface areas by the post-treatment process. Furthermore, mechanical surface treatments may create nanocrystalline layers at the top surface that are characterized by their outstanding strength. The phenomenon of surface nanocrystallization is related to severe plastic deformation which is—beside the material state prior to the mechanical post treatment—predominantly affected by the strain induced by the mechanical impact and the repetitive deformation (coverage) [31]. Various mechanical surface treatments have been proposed for surface nanocrystallization as e.g. conventional shot peening and deep rolling or air blast shot peening and ultrasonic shot peening [32, 33]. However, the generation and the analysis are outside the scope of our work presented here and hence will not be further discussed.

For transferring the established mechanical surface treatments to medical applications, corrosion cracking and fretting fatigue under a corrosive environment will be of special interest. In this respect mechanical surface treatments are well known for their beneficial impact. Cracking fatigue can be sustainably improved by means of mechanical surface treatments that prevent stress corrosion cracking, e.g. for shot peening of austenitic stainless steels [34]. For aviation applications, Lee et al. reported about the improvement of fretting fatigue behavior of shot-peened Ti6Al4V under sea water environment compared to the unpeened state [35]. Here, the compressive residual stresses induced by the shot peening are considered to be the major reason for the enhanced corrosion fatigue behavior.

To date, mechanical surface treatments are not a standard procedure for the improvement of mechanically critical implant parts, even though their potential might be high. This fact has several practical reasons: on one hand, the know-how of such a treatment is held by specialized manufacturers and the process parameters are kept strictly under lock and key. On the other hand, the reproducible outcome of such a treatment is not only dependent on the material used, but also on the handling and experience of the operator. This is why most implant manufacturers do not have direct access to this technology. However, there are some revision hip replacement systems which are put under a final shot peening or deep rolling step in the manufacturing process [28, 29]. Therefore the aim of this study was to give a substantial and practical overview and comparison on relevant mechanical treatments for medical applications of the titanium standard alloy, Ti6Al4V, in terms of fatigue strengths. With the process specializations in mind, the appropriate parameter choice for each treatment was up to the empirical experience of the industrial operators which is, in the authors’ opinion, the most practical approach.

## Materials and Methods

Hour-glass shaped rotationally symmetric fatigue specimens with a minimal diameter of 10 mm at the relieved middle section were machined from a Ti6Al4V bar according to ISO 5832–3. The material composition is given in Table 1.

**Table 1:** Chemical composition according to ISO 5832–3 and the investigated Ti6Al4V specimens.

	**Element (wt.-%)**	**C**	**V**	**Al**	**O**	**N**	**H**	**Fe**	**Ti**
ISO 5832–3	Min		3.5	5.5					
	Max	0.08	4.5	6.75	0.2	0.05	0.015	0.3	Rest
	Specimens (present study)	0.003	3.97	6.2	0.16	0.01	0.002	0.12	Rest

To remove all residual stresses introduced during turning and milling, all specimens were subjected to an annealing process at 620°C for 10 hours in an inert nitrogen environment (Table 2). Subsequently, they were bated in a 20 vol.-% nitric acid overnight at 60°C to remove the burnt layer.

**Table 2:** Annealing process.

**Annealing step**	**Parameter**
Starting temperature	40°C
Heating-up rate	44.6°C/h
Holding temperature	620°C
Exposure time after soaking	10 h
Cooling rate	17.4°C/h
Final temperature	100°C
*Processes take place under an inert nitrogen atmosphere*	

**Fig 2:**
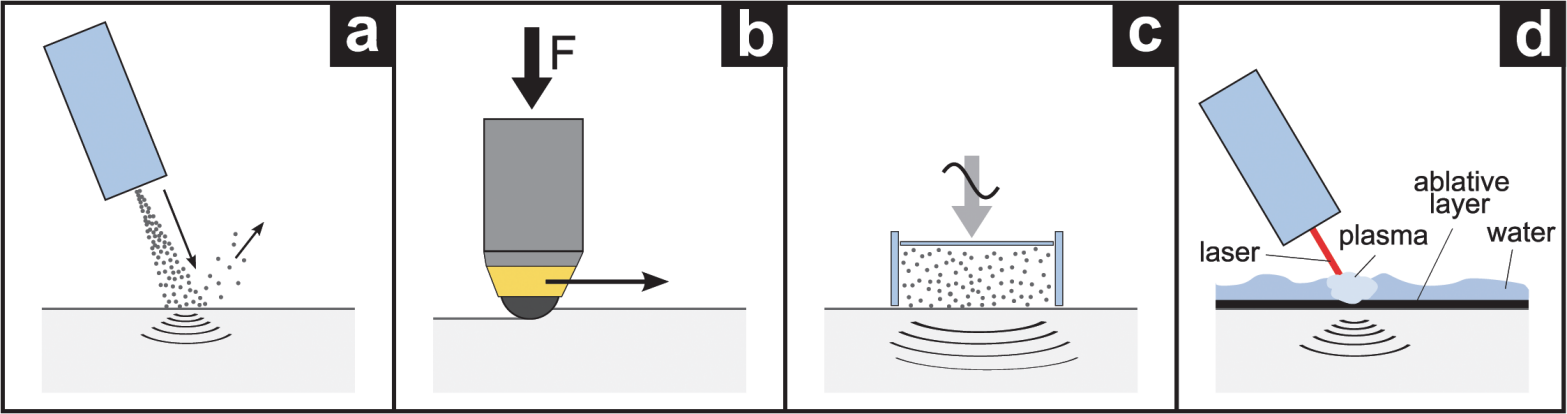
Investigated surface treatments: (a) shot peening (SP); (b) deep rolling (DR); (c) ultrasonic shot peening (US-SP); (d) laser shock peening (LSP).

Four different typical mechanical surface treatments were applied to introduce high compressive residual stresses at the surface (Fig 2). They were chosen from available processes as a result of their applicability to orthopedic implants. The parameters were chosen according to the manufacturers’ expertise in that field and with respect to the Ti6Al4V material’s state. The results of all treatments were compared to the ‘as annealed’ condition after annealing and bating.

- **Shot peening (SP)** (Fig 2A): A directed beam of the blasting material is fired on the specimen’s surface which is slowly rotated. The impacting beads locally deform the substrate at the surface introducing compressive residual stresses. Here, a combined two-step peening process is used which is identical to the application on hip revision systems and serves as a peening reference regarding fatigue performance of the other treatments.
Step 1: high-intensity, cut-wire peening (SSCW 0.4 / d = 400 μm / Almen intensity 0.012”A / coverage = 200%)
Step 2: low-intensity cleaning process using glass blasting medium to remove contaminations from the cut-wire (GP 60 / d = 60–150 μm / Almen intensity 0.006”N / coverage = 125%)
- **Deep rolling (DR)** (Fig 2B): A tool consisting of a ball or roller at the end is moved across the work piece’s surface while applying an axial force in a conventional turning machine. Under the high contact stresses, the material starts to flow and smoothens the surface while introducing residual compressive stresses. The specimens were treated with an HG6 tool (hard material sphere, d = 6 mm) (Ecoroll AG, Celle, Germany) in a conventional turning machine (speed = 530 rpm, feed rate = 0.1 mm/rev) at a pressure of 300 bar.
- **Ultrasonic shot peening (US-SP)** (Fig 2C): The principle of impacting beads and deformation of the substrate’s surface follows the classical shot peening process. The difference lies in how the ceramic beads (d = 1.0–1.2 mm, hardness = 1200 HV) are applied. This is carried out by a closed acoustic block where the beads are placed under an ultrasonic frequency (amplitude = 80 μm, peening intensity = F20.12A) for 120 seconds, forming a ‘bead gas’. During application, the treated specimen is slowly rotated (speed = 3 rpm, coverage > 125%).
- **Laser shock peening (LSP)** (Fig 2D): Generally, the substrate to be treated is covered with an opaque ablative layer (black paint) prior to the treatment. Here, no ablative layer is used to decrease and adapt the process’ impact to the small cross-section of the specimens. A high-energy laser (spot size = 3x3 mm, performance = 10 GW/cm2, pulse length = 8 ns, coverage = 100%) is fired to generate an expanding plasma which applies pressure pulses of about one million pounds per square inch, sending shock waves through the specimen. In a final step, a shot peening cleaning process using glass beads (same as for the combined shot peening process (a.)) is applied to remove burnt residuals from the laser application.

**Fig 3:**
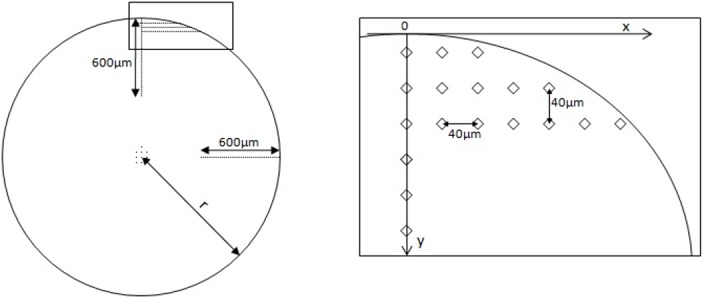
Microhardness measurement distributions in the cross-section.

To determine the effect of the mechanical surface treatments, the microstructure of the cross-section’s near-surface region was analyzed using a light microscope (Axiovert 200 MAT, Zeiss, Jena, Germany) at magnifications of up to 1000x. Etching was carried out according to Kroll and Weck [36]. On the same cross-sections, Vickers hardness measurements (test load = 25 g, dwell time = 15 s) were performed at different depths according to DIN EN ISO 6507–1 (Shimadzu HV-2000, Columbia, USA). The hardness tests were carried out on the cross-section using a distance between adjacent indentations of 40 μm to determine a hardness depth distribution with sufficient statistics (Fig 3).

Roughness measurements (Ra, Rz) on the treated surfaces were performed four times at different locations on each specimen (MahrPerthometer M2, Germany) and compared to the untreated (as annealed) condition. Measurements were carried out according to ISO 4287 (Ra, Rz) and ISO 4288 (cut-off length and length of measurement).

After each treatment, one specimen was used for the determination of residual stress depth profiles. This was realized by an iterative electrochemical layer removal and X-ray diffraction stress analysis on the newly generated surfaces. X-ray stress analysis was carried out with a diffractometer (XRD3000 PTS, Rich. Seifert Ltd., Germany) on the {114} planes of the α-titanium phase using Fe-filtered Co-Kα radiation according to the sin^2^ψ-method and the diffraction elastic constants (DEC) ½s_2_ = 11,01 10^–6^ MPa^-1^ans s_1_ = -2,61 10^–6^ MPa^-1^ (calculated according to Kröner model) [37]. 13 different tilt angles between -60° < ψ < 60° with step sizes being equidistant to sin^2^ψ were applied. The region irradiated by the X-ray beam was defined by a pinhole collimator with a nominal diameter of 1 mm. A secondary aperture with a 2.5 mm wide symmetrizing slit according to Wolfstieg was used [38]. The residual stresses were determined in axial (along the specimen) and transversal (tangential) directions. In terms of a depth analysis, a circular area with a diameter of approximately 10 mm was electrochemically removed before each measurement using the A3 electrolyte (Struers A/S, Denmark) consisting of 70% methanol, 10% 2-butoxyethanol, 3% perchloric acid and 17% water. Stress relaxation and redistribution due to material removal were neglected during stress calculation. The fitting of the X-ray interference lines was carried out using a Pearson VII function.

**Fig 4:**
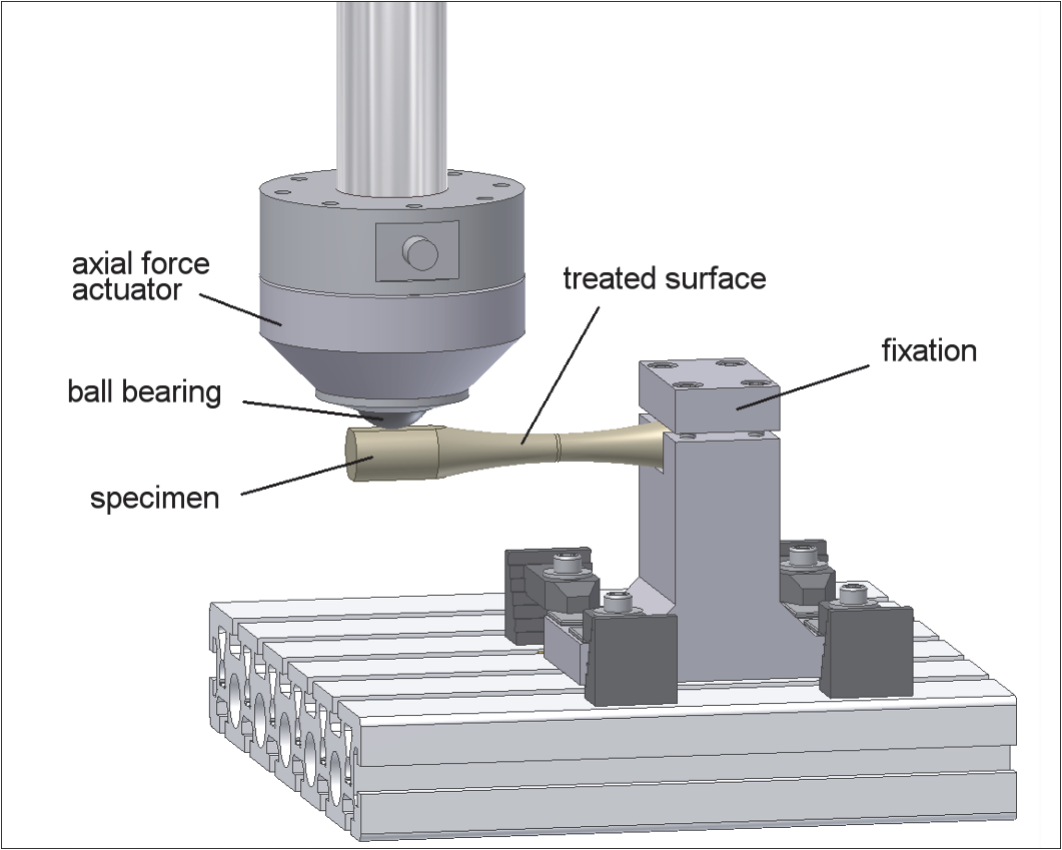
Setup for fatigue testing up to 10^7^ cycles.

Finally, specimens were subjected to a destructive fatigue testing at different load levels using a single-station servo-hydraulic uniaxial test machine (Bosch Rexroth, Germany) with lateral force compensation (Fig 4). A constant axial load ratio R = F_max_/F_min_ of 0.1 was used while tests were run under pulsating conditions (pure bending) at 10 Hz for a maximum of 10^7^ cycles (high-cycle fatigue testing) or until fracture occurred (as annealed: n = 16; SP: n = 12; DR: n = 10; US-SP: n = 10; LSP: n = 11).

## Results

**Fig 5:**
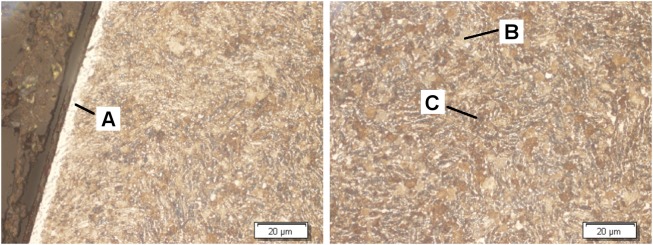
Micrograph of the cross section of the ultrasonic shot peened sample, Kroll &Weck etchant. Left: surface near region with indication of a thin α-case at the very surface (A), right: core of the sample with α -phase (B) and α +β-phase (C).

The microstructure analyses indicate that after the stress relief heat treatment, the material shows a bimodal structure consisting of α+β-phases (Fig 5). The white-brownish regions indicate a pure α -phase while the gray-bluish regions indicate the α +β two-phase regions. A change of the microstructure due to the subsequent mechanical surface treatments was not observed for any of the samples.

**Fig 6:**
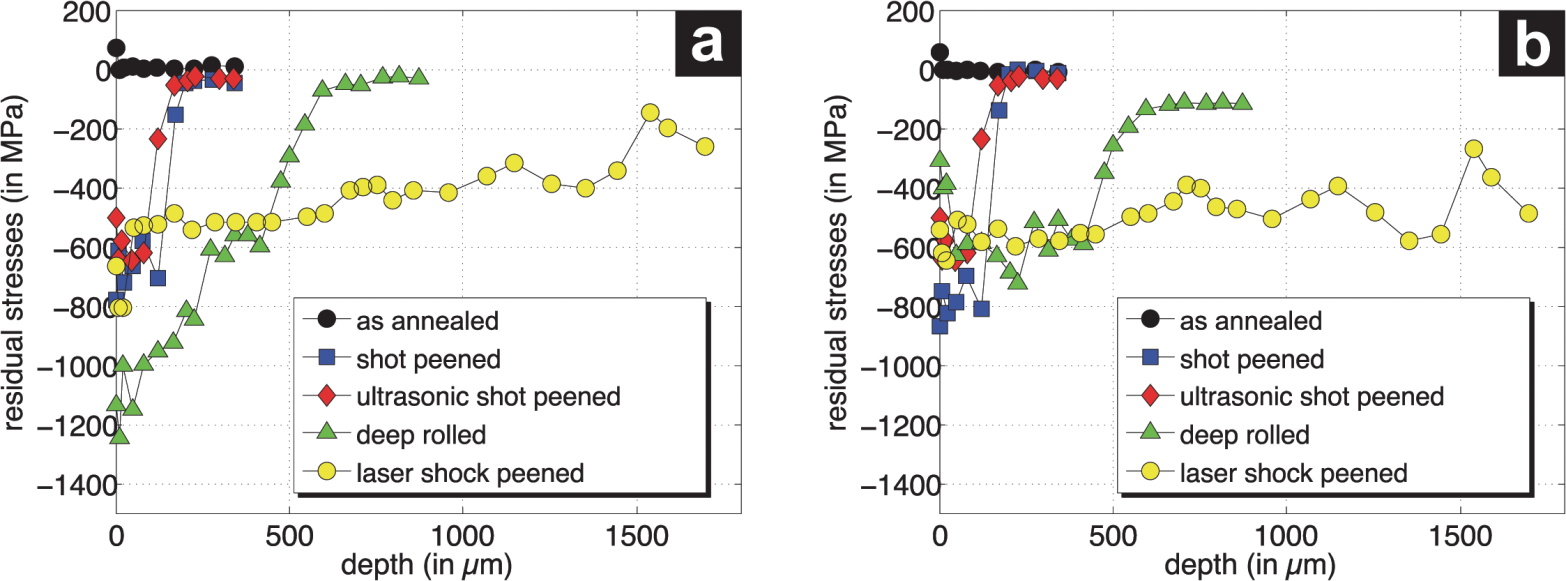
Residual stress depths profiles (a) axial direction (b) tangential (hoop) direction.

However, the Kroll &Weck etchant revealed that a 5 μm thick layer appears directly at the surface. This can be assigned to a so called α -case (hard and brittle surface layer) which might be built during the annealing treatment. Obviously, the bating process was not sufficient enough to remove the layer completely. The α -case is sensitive to crack initiation and might reduce the lifetime endurance. Due to the fact that this is the initial condition for all applied mechanical surface treatments and that the benefit of the treatment and their relative divergences are of further relevance, the thin α -case layer has no significant impact on the results. As expected, the X-ray stress analysis results show no residual stresses after annealing and bating, representing the initial condition prior to the various mechanical treatments (Fig 6). However, the residual stress values at the utmost surface reflects the values for the α -case layer. Residual stresses due to mechanical treatment are introduced as a superposition of plastic deformation and Hertzian stresses. The residual stress depth profiles show clear differences between the various mechanical surface treatments regarding the maximum residual stresses and the penetration depth. While the deep rolling process introduces very high compressive residual stresses (> 1,200 MPa) in the axial direction near the surface, other treatments show comparable maximal residual stresses around 800 MPa. As expected, the deep rolling results in a residual stress state that strongly depends on the rolling direction, meaning that much higher compressive residual stresses are induced in axial direction compared to the tangential direction. Shot peening and laser shot peening result in slightly higher compressive residual stresses in the axial direction for near-surface regions. Only for ultrasonic shot peening was an orientation-independent residual stress state determined. Based on the coverage of the compressively stressed region for laser shock peening of Ti6Al4V using the process parameters chosen by the executing company, the highest range exceeding depth of 1.6 mm was determined. In case of the deep rolling process, depths of about 600 μm are observed. However, shot peening and ultrasonic shot peening result in the lowest coverages of the compressive residual stresses of about 200 μm for both treatments.

**Fig 7:**
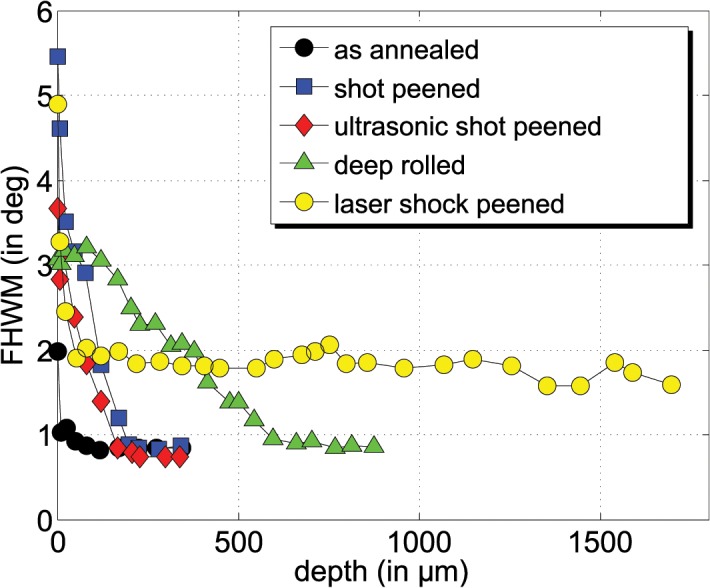
Full width at half maximum (FWHM) depth profiles (arithmetic average).

**Fig 8:**
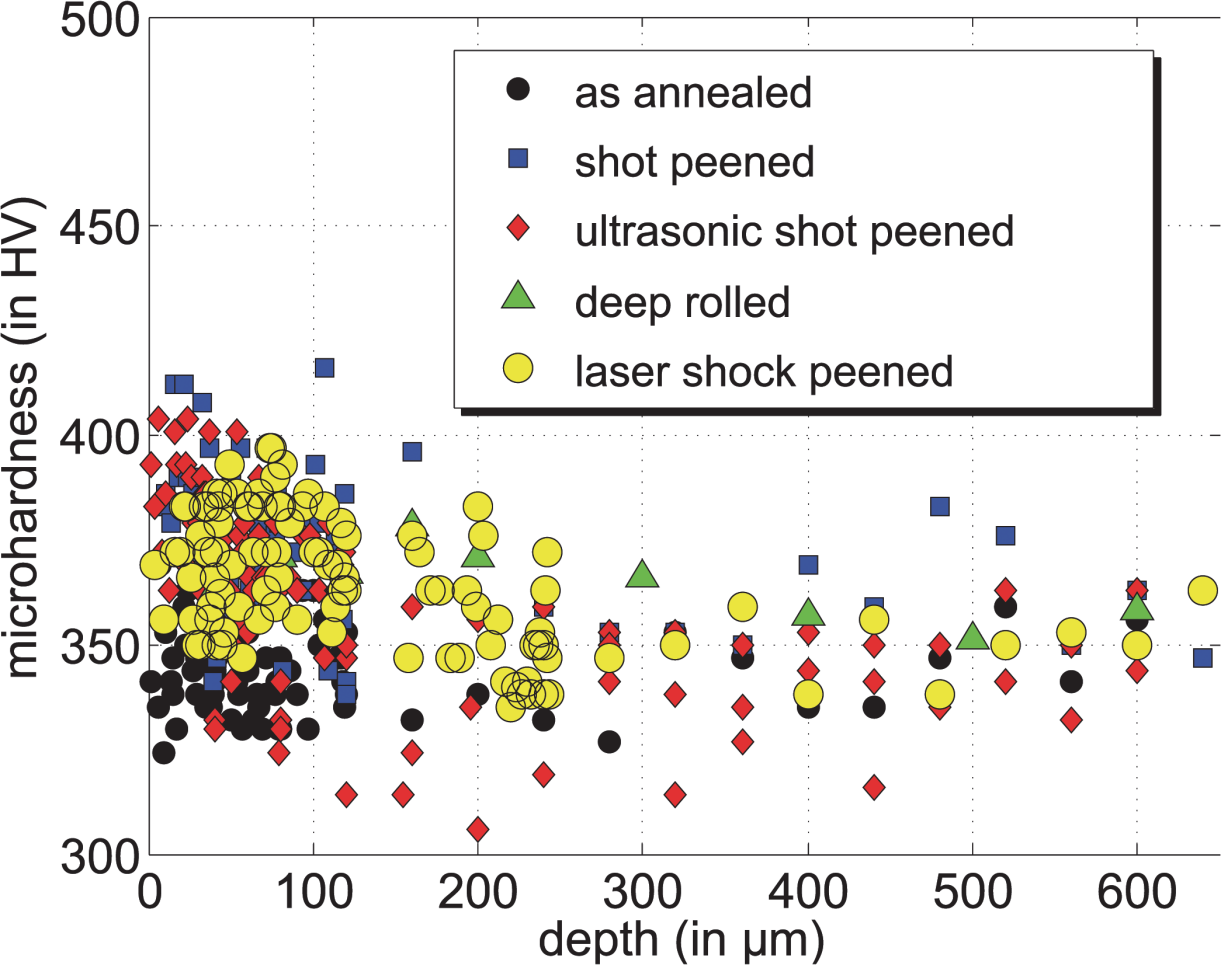
Exemplary comparison of microhardness depth profiles of shot peened with laser shot peened state.

The statements made for the coverage of the compressive residual stresses are underlined by the course of the average full width at half maximum values (FWHM) of the X-ray diffraction profiles (Fig 7). Using the identical measurement set-up for the comparative X-ray analysis, the change of the average FWHM values of the analyzed X-ray interference profiles can be used as a measure of the cold forming induced by the mechanical surface treatment. Apart from the instrument effect, the peak broadening reflects the average size of the coherent scattering regions. Here the change in the peak width is, in most cases, due to changes in the dislocation density. Due to the fact that the FWHM values were much more sensitive to the induced work hardening than the microhardness measurements, which showed a relative large scatter of the data (Fig 8), only the FWHM can be used for interpretation.

The range of compressive residual stresses corresponds well to the region where much higher FWHM values were determined due to the mechanical treatment. For higher depths, i.e. for shot peening and ultrasonic shot peeing for depths larger than 200 μm and for deep rolling for depths larger than 600 μm, the FWHM values reach the level of the initial state after annealing and bating. In contrast, the FWHM determined for the laser shot peening process is clearly higher than those of the initial material state even for large depths. This indicates that the impact of the laser shot peening might exceed the depth of 1700 μm covered by the X-ray stress analysis.

**Fig 9:**
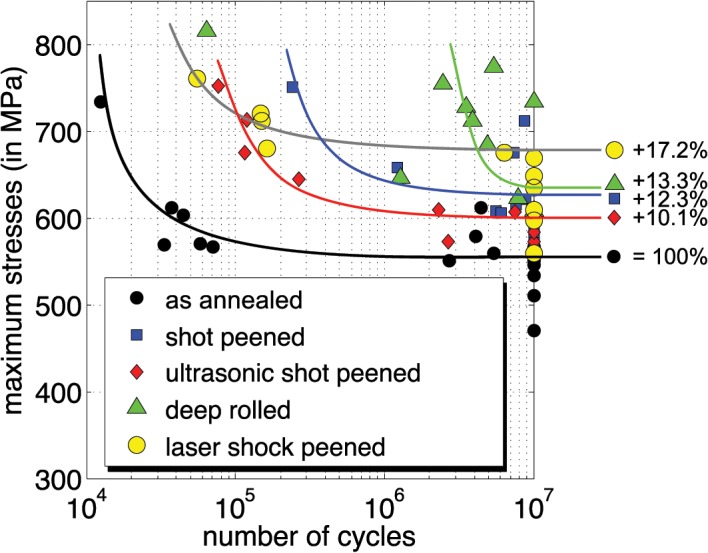
Fatigue testing results shown in a Woehler graph.

In a Woehler graph (Fig 9), the fatigue performance of the specimens after treatment is displayed. Each measurement point corresponds to a failed specimen except those which reached the total number of 10^7^ cycles, representing specimens without any rupture. The fatigue strength of a cohort is then defined as the asymptotic stress at 10^7^ cycles (high fatigue). The maximum (bending) stress at the surface varies with the external axial load that is applied during testing. All treatments result in an increase in fatigue strength of at least 10% relative to the specimens without treatment (as annealed), whereas laser shock peening showed the highest fatigue strength of all investigated treatments (+17.2%).

**Fig 10:**
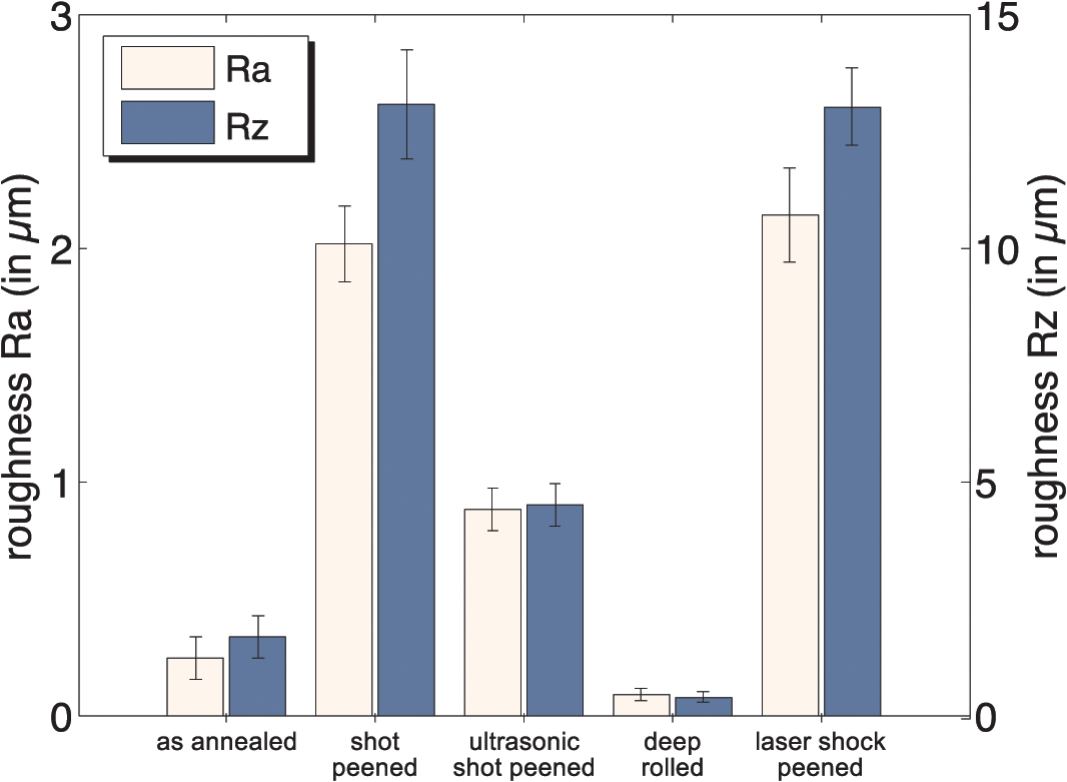
Mean roughness results of the treated surfaces.

Shot peening and laser shock peening increased the mean roughness R_a_ (R_z_) of the shot peened and laser peened surfaces by over 720% (650%) when compared to the ‘as annealed’ condition (p < 0.01) (Fig 10). Deep rolling was the only treatment to decrease the mean roughness by 64% (77%) relative to the situation after annealing and bating.

## Discussion

All of the investigated surface treatments (shot peening, deep rolling, ultrasonic shot peening and laser shock peening) have increased the high cycle fatigue strength of a Ti6Al4V standard alloy by more than 10% and have proven to be powerful tools for mechanical alterations of an implant without changing its design or material. Relative to the reference of the two-step shot peened specimens, increases in fatigue strength were found after deep rolling and laser shock peening. Various publications support these findings for Ti-base alloys after mechanical surface treatments. Schuh et al. were investigating different processes for the application on revision stems in total hip arthroplasty prior to sterilization. Shot peening has been reported as one possible treatment to strengthen a metal taper made of a Ti6Al4V alloy in a two-step treatment of cut wire peening and a subsequent glass bead blasting to remove steel contamination from the surface [28]. This method is successfully applied to the morse taper junctions on modular hip stems (MRP-Titan, Peter Brehm GmbH, Weisendorf, Germany). Surface contamination experienced during the mechanical treatment is an important issue as it may provoke an adverse mechanical or biological reaction such as residual ferrous particles from the cut wire. The limits for contamination are regulated in the standards for surgical implants, such as the EN 12010, and need to be respected prior to application. Thus, the final shot peening process using glass beads that have been applied subsequently to the shot peening by cut wire and the laser shock peening may be one option for cleaning when it is guaranteed that glass contamination is minimized to avoid increased wear due to hard foreign particles (third body wear) [39, 40]. Deep rolling was proposed as a suitable alternative to the peening treatment on a medical grade Ti6Al7Nb alloy resulting in beneficial residual stress depth distributions with its maximum of compressive residual stresses below the surface and no contaminations at the surface [29]. However, since the basic loading mode of the hip junction is bending, the highest compressive residual stresses are expected to minimize crack initiation at the very surface. Consequently, a two-step deep rolling procedure is proposed that first uses a large diameter ball followed by a deep rolling with a small diameter ball to tailor the residual stress depth distribution for exhibiting the maximum at the very surface.

In contrast, detrimental effects have also been reported on the fatigue strength of titanium alloys after mechanical surface treatment. The loss of high cycle strength after shot peening of a Ti6Al4V alloy was attributed to an over-peening effect that results in a cyclic softening of the shot peened material during fatigue testing [41]. Ludian et al. investigated the fatigue behavior of various Ti-base alloys with respect to heat treatment, environment and residual stress level induced by mechanical surface treatment like shot peening and deep rolling [42]. It has been shown that a final heat treatment resulted in a decrease in fatigue strength, which can be attributed to the anomalous mean stress sensitivity exhibited by titanium base alloys under certain heat treatment conditions in combination with the characteristic residual stress depth distributions induced by the mechanical surface treatment. Additionally, corundum grit as well as sand blasting were reported to decrease the endurance limit by up to 35–40% compared to polished samples, which was explained by residual corundum particles in the surface near region and small surface defects that act as crack initiators during cyclic loading [43, 44].

From a practical point of view, the flexibility of these surface treatments, the associated implementation in the manufacturing process and the economics need to be considered, as the treatments are quite different. While shot peening or laser shock peening are mainly robot-controlled and flexible in their application, deep rolling as well as ultrasonic shot peening need more effort to be adapted to complex geometries. This may, however, not become relevant for an appropriately high number of treated components. In addition, the surface roughness that is adjusted by the treatment should not have an adverse effect on the component’s function, e.g. in modular total hip arthroplasty [45].

This study has investigated the pure mechanical outcome of surface treatments and does not account for a complex physiological environment. This includes a multi-axial load pattern, micromotions between assembled components (fretting) as well as a corrosive environment, even though the latter does not seem to have a negative influence on the mechanical outcome after shot peening [46]. King et al. have shown an increase in fretting resistance after shot peening and laser shock peening of a Ti6Al4V alloy [47]. Based on the reported results, the fretting protective performance under relevant corrosive and loading conditions may be further investigated under a more physiological environment. In addition, the present study has been limited to the standard titanium alloy, Ti6Al4V. Other implant materials, such as CoCr alloys, other titanium alloys, stainless steels or even ceramics may behave differently and need to be considered separately. Other relevant methods which strengthen the material prior to sterilization may also be considered. As a new method, abrasive water jet peening was proposed for mechanical surface treatment of metal orthopedic implants [48]. The peening process induces biaxial compressive residual stresses for Ti6Al4V of up to -400 MPa and the stress concentrations factor is decreased. It has been reported that the components’ fatigue strength by the abrasive water jet peening as well as the average surface roughness (R_a_ up to 14.2 μm) is significantly increased. Additionally, cavitation shotless peening may also be interesting from a fatigue standpoint as well as a contamination point of view [49].

## Conclusion

Mechanical surface treatments are not among the standard processing techniques for implants used in orthopedic applications or in trauma surgery. This comprehensive comparison of relevant mechanical treatments has shown their potential, as all investigated treatments increased the fatigue performance of the Ti6Al4V alloy and by up to 17.2% in the case of combined laser shock and glass shot peening processes. This is the first study to directly compare relevant surface treatments. The findings should encourage manufacturers to consider this option, especially against the background of small and bone-conserving implants for load-bearing applications whose fracture risk is increased.

## References

[1] Bothe RT, Beaton LE, Davenport HA. Reaction of bone to multiplemetallic implants. Surg Gynecol Obstet. 1940;71: 598–602.

[2] Branemark PI. Osseointegration and its experimental background. J Prosthet Dent. 1983;50: 399–410. 6352924 10.1016/s0022-3913(83)80101-2

[3] Leventhal GS. Titanium, a metal for surgery. J Bone Joint Surg Am. 1951;33: 473–474. 14824196

[4] Grupp T, Weik T, Bloemer W, Knaebel HP. Modular titanium alloy neck adapter failures in hip replacement—failure mode analysis and influence of implant material. BMC Musculoskelet Disord. 2010;11: 3 10.1186/1471-2474-11-3 20047653 PMC2824687

[5] Banovetz JM, Sharp R, Probe RA, Anglen JO. Titanium Plate Fixation: A Review of Implant Failures. J Orthop Trauma. 1996;10: 389–394. 8854316 10.1097/00005131-199608000-00005

[6] Alvarez DB, Aparicio JP, Fernández EL, Múgica IG, Batalla DN, Jiménez JP. Implant breakage, a rare complication with the Gamma nail. A review of 843 fractures of the proximal femur treated with a Gamma nail. Acta Orthop Belg. 2004;70: 435–443. 15587032

[7] Atwood SA, Patten EW, Bozic KJ, Pruitt LA, Ries MD. Corrosion-Induced Fracture of a Double-Modular Hip Prosthesis: A Case Report. J Bone Joint Surg Am. 2010;92: 1522–1525. 10.2106/JBJS.I.00980 20516330

[8] Busch CA, Charles MN, Haydon CM, Bourne RB, Rorabeck CH, MacDonald SJ, et al Fractures of distally-fixed femoral stems after revision arthroplasty. J Bone Joint Surg Br. 2005;87-B: 1333–1336. 10.1302/0301-620X.87B10.16528 16189303

[9] Dangles CJ, Altstetter CJ. Failure of the Modular Neck in a Total Hip Arthroplasty. J Arthroplasty. 2010;25: 1169.e1165–1169.e1167. 10.1016/j.arth.2009.07.015 19837558

[10] Lakstein D, Eliaz N, Levi O, Backstein D, Kosashvili Y, Safir O, et al Fracture of Cementless Femoral Stems at the Mid-Stem Junction in Modular Revision Hip Arthroplasty Systems. J Bone Joint Surg Am. 2011;93: 57–65. 10.2106/JBJS.J.01770 21209269

[11] Magnissalis EA, Zinelis S, Karachalios T, Hartofilakidis G. Failure analysis of two Ti-alloy total hip arthroplasty femoral stems fractured in vivo. J Biomed Mater Res B Appl Biomater. 2003;66B: 299–305. 12808587 10.1002/jbm.b.10003

[12] Patel A, Bliss J, Calfee RP, Froehlich J, Limbird R. Modular Femoral Stem-Sleeve Junction Failure After Primary Total Hip Arthroplasty. J Arthroplasty. 2009;24: 1143.e1141–1143.e1145. 10.1016/j.arth.2008.09.006 18835691

[13] Sotereanos NG, Sauber TJ, Tupis TT. Modular Femoral Neck Fracture After Primary Total Hip Arthroplasty. J Arthroplasty. 2012;28: 196.e197–e199. 10.1016/j.arth.2012.03.050 22658428

[14] Iwakura T, Niikura T, Lee SY, Sakai Y, Nishida K, Kuroda R, et al. Breakage of a third generation gamma nail: a case report and review of the literature. Case Rep Orthop. 2013: 1–5. 10.1155/2013/172352 PMC3671522 23762698

[15] Yukata K, Doi K, Hattori Y, Sakamoto S. Early Breakage of a Titanium Volar Locking Plate for Fixation of a Distal Radius Fracture: Case Report. J Hand Surg Am. 2009;34: 907–909. 10.1016/j.jhsa.2009.01.004 19410996

[16] Graves S, Davidson D, de Steiger R, Tomkins A, Ryan P, Griffith L, et al Australian National Joint Replacement Registry—Annual Report. Adelaide: Australian Orthopaedic Association 2012.

[17] Garellik G, Karrholm J, Rogmark C, Rolfson O, Herberts P. Swedish Hip Register—Annual Report. Gothenburg: University of Gothenburg 2011.

[18] Hsu CC, Yongyut A, Chao CK, Lin J. Notch sensitivity of titanium causing contradictory effects on locked nails and screws. Med Eng Phys. 2010;32: 454–460. 10.1016/j.medengphy.2010.03.006 20430681

[19] Sul YT, Johansson CB, Petronis S, Krozer A, Jeong Y, Wennerberg A, et al Characteristics of the surface oxides on turned and electrochemically oxidized pure titanium implants up to dielectric breakdown:: the oxide thickness, micropore configurations, surface roughness, crystal structure and chemical composition. Biomaterials. 2002;23: 491–501. 11761170 10.1016/s0142-9612(01)00131-4

[20] Bergmann G, Graichen F, Rohlmann A. Hip joint contact forces during stumbling. Langenbecks Arch Surg. 2004;389: 53–59. 14625775 10.1007/s00423-003-0434-y

[21] Ritchie RO, Davidson DL, Boyce BL, Campbell JP, Roder O. High-cycle fatigue of Ti-6Al-4V. Fatigue Fract Eng M. 1999;22: 621–631.

[22] Mehran N, North T, Laker M. Failure of a modular hip implant at the stem-sleeve interface. Orthopedics. 2013;36: e978–e981. 10.3928/01477447-20130624-33 23823060

[23] Sivash KM. The development of a total metal prosthesis for the hip joint from a partial joint replacement. Reconstr Surg Traumatol. 1969;11: 53–62. 4886894

[24] Wilson DAJ, Dunbar MJ, Amirault JD, Farhat Z. Early Failure of a Modular Femoral Neck Total Hip Arthroplasty Component: A Case Report. J Bone Joint Surg Am. 2010;92: 1514–1517. 10.2106/JBJS.I.01107 20516328

[25] Wright G, Sporer S, Urban RM, Jacobs J. Fracture of a Modular Femoral Neck After Total Hip Arthroplasty: A Case Report. J Bone Joint Surg Am. 2010;92: 1518–1521. 10.2106/JBJS.I.01033 20516329 PMC2874667

[26] Gilbert JL, Buckley CA, Jacobs JJ. In vivo corrosion of modular hip prosthesis components in mixed and similar metal combinations. The effect of crevice, stress, motion, and alloy coupling. J Biomed Mater Res.1993;27: 1533–1544. 8113241 10.1002/jbm.820271210

[27] Kurtz SM, Devine JN. PEEK biomaterials in trauma, orthopedic, and spinal implants. Biomaterials. 2007;28: 4845–4869. 17686513 10.1016/j.biomaterials.2007.07.013 PMC2040108

[28] Schuh A, Uter W, Holzwarth U, Kachler W, Goske J, Zeiler G. Comparative surface examinations of morse taper junctions of the MRP-Titan stem shot peened with glas beads. Biomed Tech (Berl). 2004;49: 334–339. 15655925 10.1515/BMT.2004.062

[29] Schuh A, Zeller C, Holzwarth U, Kachler W, Wilcke G, Zeiler G, et al Deep rolling of titanium rods for application in modular total hip arthroplasty. J Biomed Mater Res B Appl Biomater. 2006;81B: 330–335. 10.1002/jbm.b.30669 16969829

[30] Ludian T, Wagner L. Mechanical Surface Treatments for Improving Fatigue Behavior in Titanium Alloys. Adv Mater Sci. 2008;8: 44–52.

[31] Umemoto M. Nanocrystallization of Steels by Severe Plastic Deformation. Mater Trans. 2003;44: 1900–1911.

[32] Altenberger I, Scholtes B, Martin U, Oettel H. Cyclic deformation and near surface microstructures of shot peened or deep rolled austenitic stainless steel AISI 304. Mater Sci Eng. 1999;264: 1–16.

[33] Umemoto M, Todaka Y, Tsuchiya K. Formation of Nanocrystalline Structure in Steels by Air Blast Shot Peening. Mater Trans. 2003;44: 1488–1493.

[34] Friske WH, Page JP. Shot peening to prevent the corrosion cracking of austenitic stainless steels. JMES. 1979;1: 20–32.

[35] Lee H, Mall S, Allen WY. Fretting fatigue behavior of shot-peened Ti–6Al–4V under seawater environment. Mater Sci Eng. 2006;420: 72–78.

[36] Petzow G, Carle V. Metallographisches, keramographisches, plastographisches Ätzen. Borntraeger; 2006. p. 298.

[37] Macherauch E, Mueller P. Das sin^2^psi-Verfahren der röntgenographischen Spannungsmessung. Z Angew Phys. 1961;13: 305–312.

[38] Wolfstieg U. Die Symmetrisierung unsymmetrischer Interferenzlinien mit Hilfe von Spezialblenden. HTM Härterei-Techn Mitt. 1976;31: 23–27.

[39] Schuh A, Holzwarth U, Kachler W, Goske J, Zeiler G. Surface Characterisation of Shot Peened Implants with Glas Beads in Total Hip Arthroplasty. Zentralbl Chir. 2004;129: 225–229. 15237332 10.1055/s-2004-822740

[40] Schuh A, Uter W, Holzwarth U, Kachler W, Goske J, Raab B, et al Residual Particle Free Surfaces After Shot Peening in Modular Hip Arthroplasty are Feasible. Zentralbl Chir. 2005;130: 576–579. 16382407 10.1055/s-2005-872558

[41] Wagner L, Lütjering G. Influence of shot peening treatment on the fatigue life of Ti6Al4V. Shot Peening, Am Shot Peening Soc. 1984: 201–207.

[42] Ludian T, Kocan M, Rack HJ, Wagner L. Residual-stress-induced subsurface crack nucleation in titanium alloys. Int J Mater Res. 2006;97: 1425–1431.

[43] Leinenbach C, Eifler D. Fatigue and cyclic deformation behaviour of surface-modified titanium alloys in simulated physiological media. Biomaterials. 2006;27: 1200–1208. 16140373 10.1016/j.biomaterials.2005.08.012

[44] Baleani M, Viceconti M, Toni A. The Effect of Sandblasting Treatment on Endurance Properties of Titanium Alloy Hip Prostheses. Artif Organs. 2000;24: 296–299. 10816203 10.1046/j.1525-1594.2000.06486.x

[45] Panagiotidou A, Meswania J, Hua J, Muirhead-Allwood S, Hart A, Blunn G. Enhanced wear and corrosion in modular tapers in total hip replacement is associated with the contact area and surface topography. J Orthop Res. 2013;31: 2032–2039. 10.1002/jor.22461 23966288

[46] Sonntag R, Reinders J, Gibmeier J, Jaeger S, Kretzer JP. Ti-6Al-4V Fatigue Strength After Shot Peening: The Role of a Corrosive Environment In: Mitchell MR, Smith SW, Woods T, Berg BT, editors. Fatigue and Fracture of Medical Metallic Materials and Devices. West Conshohocken: ASTM International; 2013 pp. 98–108.

[47] King A, Steuwer A, Woodward C, Withers PJ. Effects of fatigue and fretting on residual stresses introduced by laser shock peening. Mater Sci Eng A. 2006;435–436: 12–18.

[48] Arola DD, McCain ML. Abrasive waterjet peening: A new method of surface preparation for metal orthopedic implants. J Biomed Mater Res. 2000;53: 536–546. 10984702 10.1002/1097-4636(200009)53:5<536::aid-jbm13>3.0.co;2-v

[49] Soyama H, Macodiyo DO, Mall S. Compressive Residual Stress into Titanium Alloy Using Cavitation Shotless Peening Method. Tribol Lett. 2004;17: 501–504

